# YASARA Model–Interactive Molecular Modeling from Two Dimensions to Virtual Realities

**DOI:** 10.1021/acs.jcim.3c01136

**Published:** 2023-10-02

**Authors:** Kornel Ozvoldik, Thomas Stockner, Elmar Krieger

**Affiliations:** † Center for Physiology and Pharmacology, Institute of Pharmacology, Medical University of Vienna, Waehringerstr. 13A, 1090 Vienna, Austria; ‡ YASARA Biosciences GmbH, Wagramer Str. 25/3/45, 1220 Vienna, Austria

## Abstract

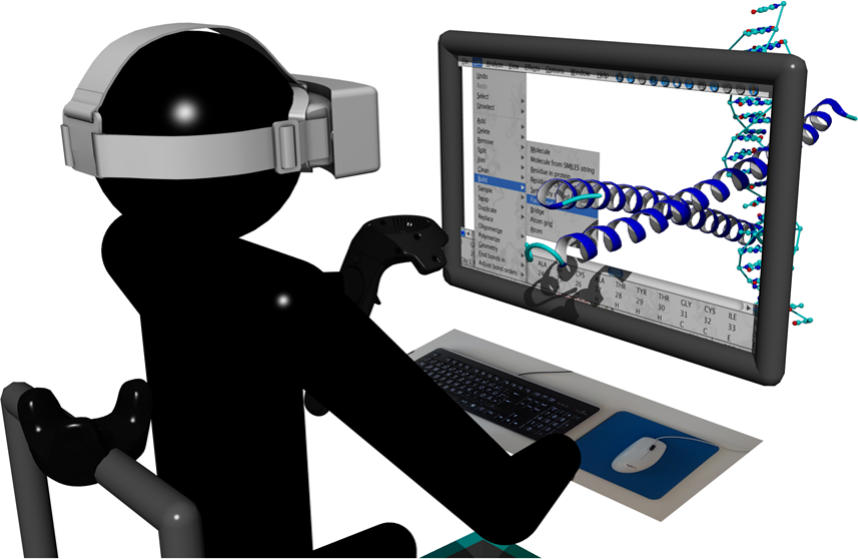

The industry’s transition from three-dimensional (3D) glasses to virtual reality (VR) headsets has left modelers stranded without hardware supply, since walking around and waving arms in a virtual world is a great experience but also very tiring when doing time-intensive modeling work. We present a novel software implementation that uses a VR headset while sitting at a desk in front of the normal screen, which is beamed into the virtual reality together with keyboard, mouse, and chair using the headset’s cameras and an extra tracker attached to the seat-back. Compared to 3D glasses, this yields a comparably relaxing but much more immersive workplace and provides additional possibilities such as taking molecules into one’s hands, standing up, and walking or teleporting through the models. This VR functionality has been combined with a molecular graphics engine based on Vulkan, a next-generation cross-platform application programming interface (API) for GPUs and the successor of the widely used Open Graphics Library (OpenGL). It is built into the YASARA Model program, which includes many features like small and large molecule builders, electron densities, partial surfaces, contact analysis, coordinate manipulation, and animations. Interactive tutorials are provided to guide modelers into VR and familiarize them with the molecular modeling features. YASARA Model is available for Linux, Windows, Android, and MacOS (the latter without VR) with an introductory video at www.YASARA.org/vr.

## Introduction

Since the implementation of interactive side-by-side stereo viewing in 1969 with a dual trace oscilloscope,^1^ molecular modeling in three dimensions has become widely accessible thanks to cheap shutter glasses, compatible monitors, and stereocapable graphics processing units (GPUs) like AMD FireGL or NVIDIA Quadro. Unfortunately, device drivers for stereo glasses like NVIDIA 3D Vision were largely abandoned during the past few years, in favor of supporting VR headsets. Consequently, Vulkan started without providing an equivalent of the OpenGL technique of using two frame buffers for each eye to generate stutter- and tearing-free stereo images dubbed quad-buffered stereo, leaving modelers stranded with obsolete hardware.

The technological jump from 3D glasses to VR headsets sparked the development of numerous scientific projects.^2^ Foundational work in the usage of modern VR headsets and controllers for molecular visualization was laid out with the Molecular Rift^3^ software package. Prominent molecular visualization and analysis desktop applications integrated VR capabilities: VMD^4^ supports the rendering of VR movies. ChimeraX,^5^ UnityMol,^6^ and Coot^7^ fully expanded their applications into VR, including VR controller utilization. A new generation of fully VR-based frameworks has entered the stage of molecular modeling: Nanome^8^ and Narupa,^9^ the latter with successful application of VR methodology in practice.^10^ Notably, ChimeraX, Nanome, and Narupa are capable of simultaneous multi-person sessions. A wide range of interactive VR applications has been developed for specific tasks: building molecules with VRChem,^11^ docking ligands to flexible receptors with DockIT,^12^ exploration of chemical space populated by DrugBank compounds with Virtual Reality Chemical Space,^13^ reactive molecular dynamics (MD) with InteraChem,^14^ MD of guest molecules in metal–organic framworks with MOF-VR,^15^ visualization of biological electron-transfer dynamics with iBET,^16^ visualizing biomolecular electrostatics with UnityMol-APBS,^17^ 3D chromatin structure modeling with CSynth,^18^ DNA modeling with Vivern,^19^ or creating mesoscale scenes of biological macromolecules with CellPAINT-VR.^20^ ProteinVR^21^ and VRmol^22^ are easily accessible web-based VR molecular viewers.

In this spectrum of VR applications, YASARA Model assumes the role of a general purpose molecular modeling software package with a large number of modeling features that combines the productivity of a desktop application with the immersiveness of VR. The current VR equipment, driven by the video gaming industry, suffers from a number of drawbacks when applied in scientific practice: Reduced precision as well as operator fatigue as a result of standing and holding controllers in midair (known as the *gorilla arm syndrome*(2)). Here we describe how YASARA Model overcomes these drawbacks to combine the best of both, real and virtual, worlds.

## Methods

### Implementation Details

The VR functionality was implemented as part of the YASARA Model program. The Linux and Windows versions support all of the VR features described here. For Android, a version based on Google cardboard with a limited number of features is available. We tested seven different VR headsets, five with outside-in tracking via two to four Lighthouse base stations (HTC Vive, Vive Pro, Vive Pro 2, Vive Cosmos Elite, Valve Index) and the Meta Quest 2 and Pico 4 with inside-out tracking through cameras on the headset.

We use the device-agnostic OpenVR API provided by SteamVR, a commercial VR software suite developed by Valve Corporation, to communicate with VR headsets. Although proprietary, SteamVR universally supports all relevant headsets. A migration to the new OpenXR standard is planned as soon as support for trackers and cameras has matured, which will allow one to selectively blend in keyboard and mouse not only on the Vive (Pro) and Index headsets but also on those (Meta Quest 2, Vive Cosmos Elite, Pico 4) that currently only support complete pass-through, a feature that directly passes front camera images to the user display.

Access to the front cameras via OpenVR works fine on Windows but is unreliable and prone to errors on Linux. Instead we gain direct access through VideoForLinux2, a widely used Linux kernel interface for video capture devices. Due to the additional overhead, frame reading and conversion are done in a separate CPU thread in parallel to the main application. Manually measuring and compensating the camera lag time for each headset allows one to present a smooth video stream to the user.

The main challenge with VR is that the application needs to redraw the view at least 90 times per second to avoid stutter and motion sickness no matter what the user does. Scientific applications commonly use one main thread (i.e., sequential CPU instructions) for the core program and start additional threads if they encounter a task that can be parallelized across multiple CPU cores. Not surprisingly, many tasks take longer than 1/90th of a second (e.g., force field parameter assignment, or one MD simulation step for a large system), delaying the drawing of the next VR view. For an application like YASARA, which has been in development for over 30 years, this might require a complete rewrite of the core architecture at first sight. Fortunately, the problem is comparable to the need to display a progress bar and keep the program responsive during longer operations, which already existed 30 years ago. A progress bar function is typically called repeatedly from the inner loop of a lengthy calculation, updates the screen in regular intervals, shows a progress bar, reads user input, and tells the calling function to stop the calculation if requested by the user. Our solution to the VR problem is to expand the progress bar function: when called for the first time at the beginning of a lengthy calculation, it creates a copy of the data structures required for visualization and starts a separate thread that uses this copy to update the VR view 90 times per second. In the mean time, the main thread can manipulate the original data structures (e.g., to create or delete molecules). When finished, it tells the VR thread to stop and takes over the VR view update again. This approach is simplified by the design of the Vulkan API, which—contrary to OpenGL—does not care which thread calls its functions.

### Displaying the Physical Keyboard and Mouse

**Figure 1:**
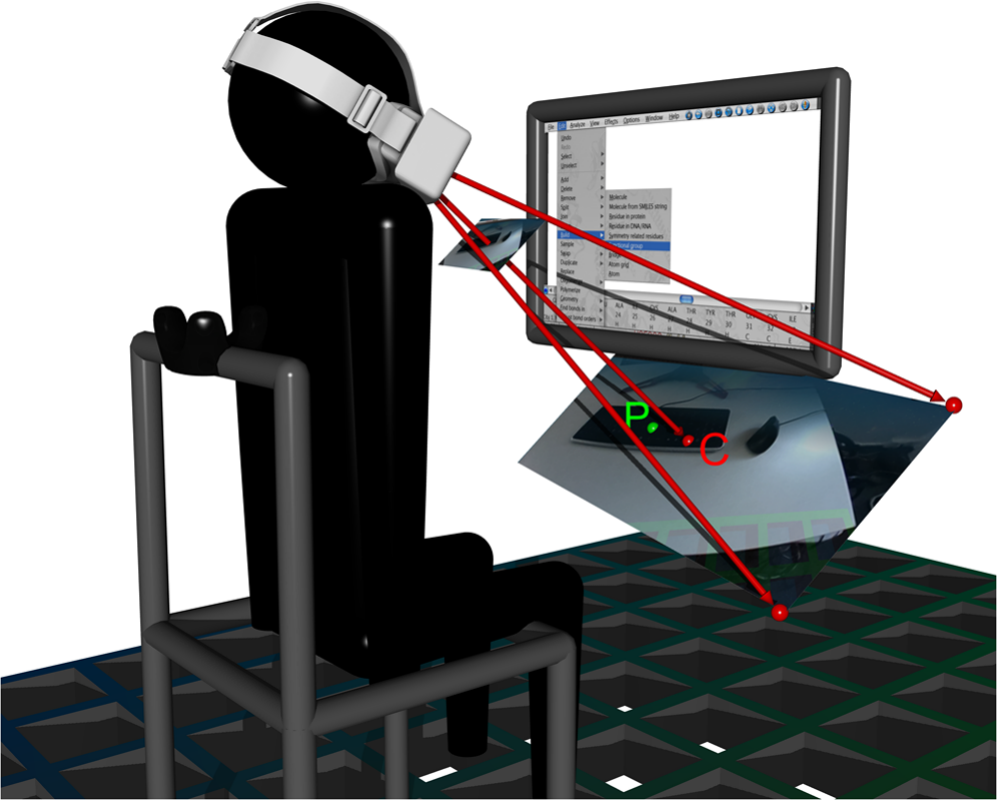
Front camera image with image center **C** is projected from the focal plane (represented by the small image in front of the headset) into the plane spanned by the camera optical axis and point **P**, located 10 cm below the bottom edge and 30 cm away from the monitor, where the keyboard is assumed to be. The position and size of the image of the keyboard are then approximately the same as of the real one. The part of the image above the monitor bottom edge is faded out via an alpha gradient.

A prerequisite for a relaxed molecular modeling work flow is the ability to use a keyboard and mouse. Instead of forcing the user to type blindly, we stream the video feed from the headset front cameras into VR. To avoid motion sickness, we are faced with the challenge to display the image of keyboard and mouse with the same position and size as the real ones, independent of the headset position and orientation. This process can be pictured as creating for each eye a billboard in VR and painting it with the image from the respective camera. Keyboard and mouse are not tracked; hence, we assume they are located at a point 10 cm below the bottom of and 30 cm away from the monitor. The virtual billboard is placed orthogonal to the camera optical axis in the same plane as the aforementioned point (Figure 1). The billboard is then resized depending on the camera distance from the plane and the focal length, read out from the camera intrinsic matrix delivered by SteamVR. Another important piece of information provided by the camera intrinsics is the shift of the camera sensor relative to the focal point. As this information is not always accurate, we allow one to manually adjust the shift to optimize the overlap between rendered controllers and their camera view, which is especially important when using two front cameras to create a stereo image. As to not unnecessarily occlude the scene with the camera image, we apply an alpha gradient across the billboard texture, which lets the image vanish above the bottom monitor edge. We also scale the alpha values depending on the distance and angle of the headset to the keyboard to smoothly blend out the image when looking or walking away.

## Results

### Molecular Modeling in VR

**Figure 2:**
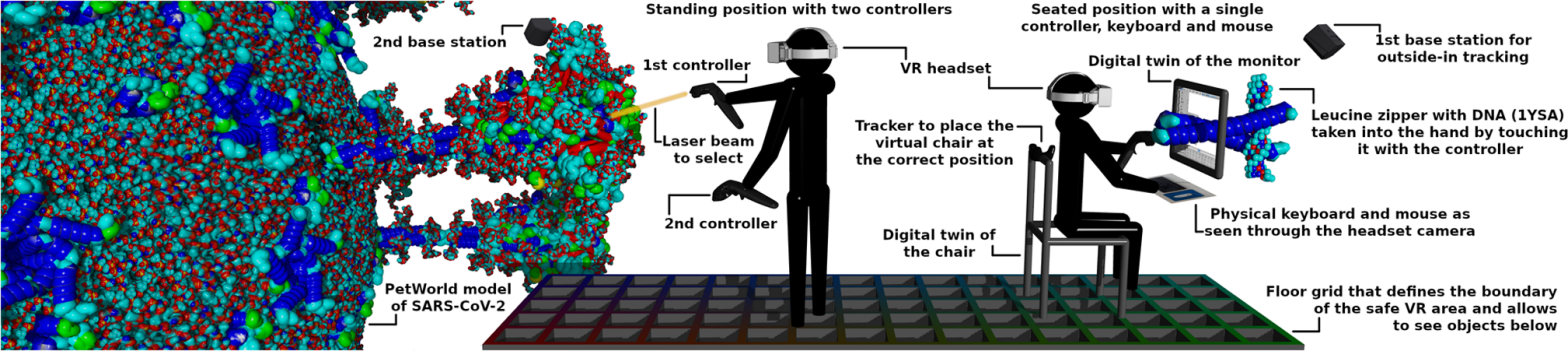
Seated position (right) with tracked chair, virtualized monitor, and blended in mouse and keyboard for productive molecular modeling using a controller as 3D mouse. Standing position (center) with two controllers for on-foot exploration of molecular models (left).

The location of the monitor in VR is configured by positioning the controllers at the four corners of the physical screen, hereby anchoring the global coordinate system in the center of the screen. After configuration, the VR controllers can be used as 3D mice, even without wearing the headset. Optionally, a Vive Tracker is placed on the chair to create a digital twin in VR. While in a seated position (Figure 2, right), the graphical user interface (GUI) is displayed on the virtual monitor and can be operated with a VR controller or mouse. If the user looks at the table in front of the screen, the physical mouse and keyboard are displayed as part of the 3D scene using the front cameras (in stereo if available) to allow for easy typing and productive molecular modeling. For headsets that allow for only complete pass-through, adjustable oversized text is shown on the real screen to facilitate typing. As front camera resolution is generally poor, we recommend using a backlit keyboard with large print. The user can stand up at any time, grab the second controller, attach the GUI to it, and walk safely inside the boundaries of the see-through floor grid for an on-foot exploration of the molecular models (Figure 2, center). Objects in the scene are manipulated by first selecting them with a laser beam emanating from the controller or by touching them with the controller directly and then gripping the controller tightly to move the objects in space. Cameras can be switched on permanently to create a mixed reality experience or to communicate with other people in the same room.

Combining the methods described above with biomolecular instancing techniques^23^ we are able to interactively visualize large scenes, e.g., a model of SARS-CoV-2 with 6 million atoms (Figure 2, left), smoothly in VR with a constant 90 frames per second on an NVIDIA GeForce RTX 4080 graphics card.

**Figure 3:**
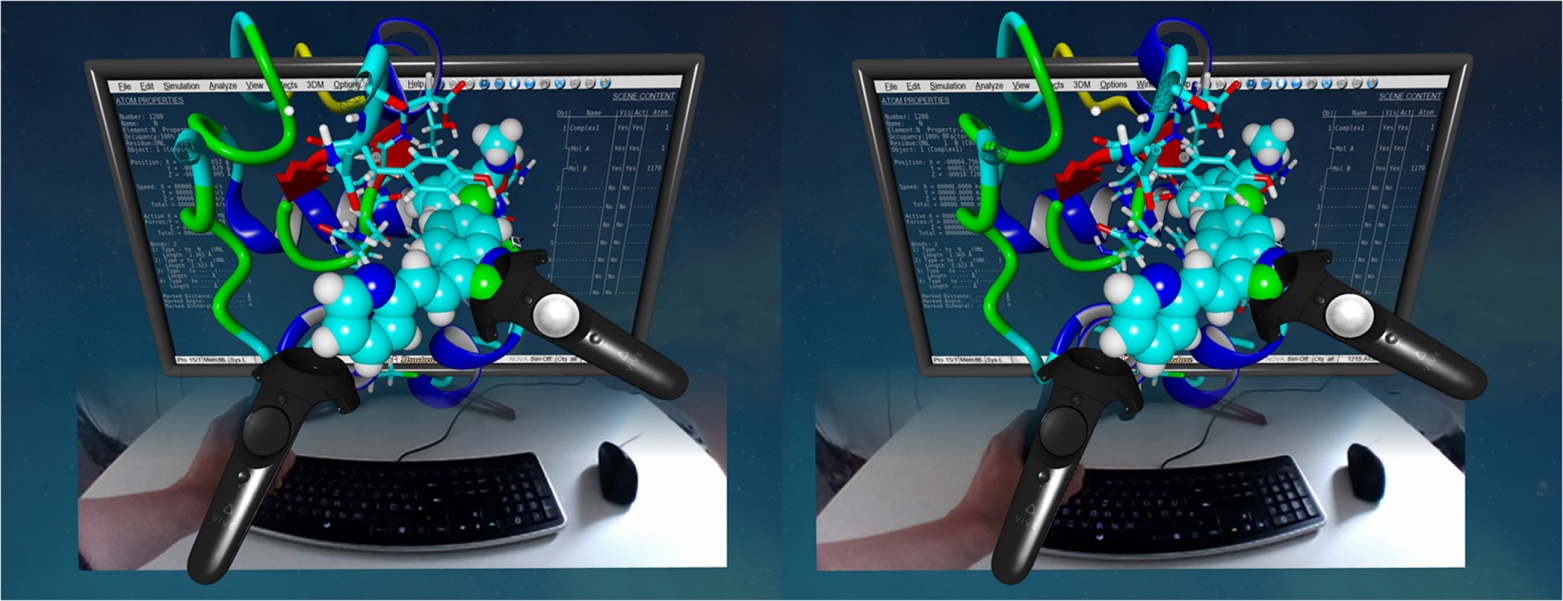
Cross-eyed stereo view of the manual docking of Axitinib to the resuscitation promoting factor B (RpfB, PDB ID: 4EMN), as part of the YASARA VR tutorial. Crossing eyes until the left and right image overlap creates the optical illusion of a single 3D image, offering a glimpse into VR. Molecules are manipulated either directly with one’s hands by grabbing the VR controllers or through a mouse and keyboard, blended in via VR headset cameras. The noticeable deformation of the keyboard vanishes when looking directly at the keyboard and is due to lens distortion, an optical effect resulting in straight lines getting curved with increasing distance from the camera image center.

**Figure 4:**
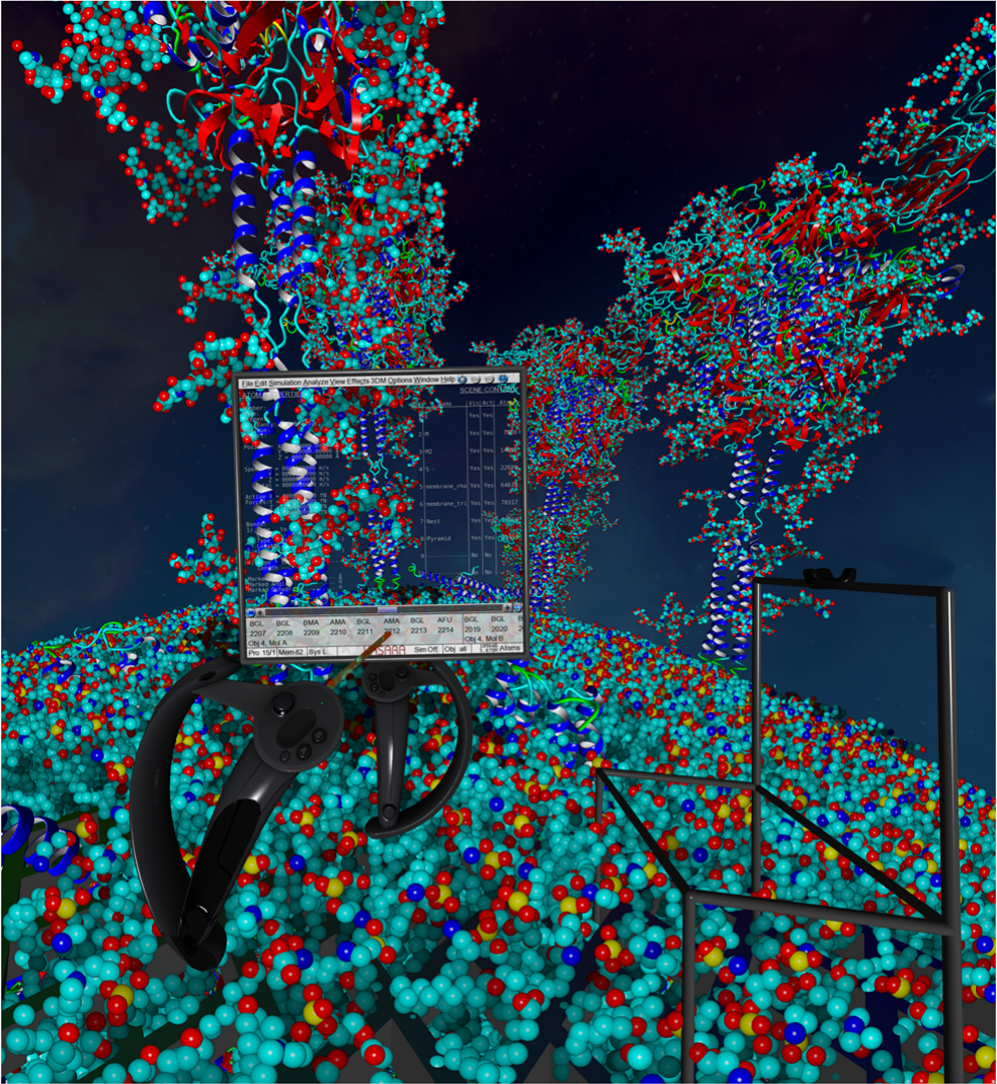
On-foot exploration in VR of an SARS-CoV-2 model (PetWorld database^23^ accession code: sarscov2, www.YASARA.org/petworld) as part of the YASARA VR tutorial. The GUI is portable and can be attached to either a controller or a selected atom. Away from the keyboard and mouse, the menu is operated using the selection beam. If a Vive Tracker is attached to one’s real chair, it appears in VR, making it easy to sit down again.

We provide an interactive tutorial to support beginners: Detailed instructions and a flowchart help users to decide which hardware to buy and how to set it up. The tutorial guides them through the first hurdles of activating their VR system, placing the screen in VR, using VR controllers as 3D mice, and finally putting on their headset to enter VR. Here the modelers find themselves in a VR playground centered around the protein AlkB (PDB ID: 3BIE) where they learn how to interact with molecules and navigate the GUI with VR controllers. After returning to their desk, the tutorial shows how to utilize mouse and keyboard while in VR to visualize protein–ligand interactions in a basic drug design example involving HIV protease (PDB ID: 3I7E). Next, the users look at the result of a virtual screening run on RpfB with several drugs approved by the Food and Drug Administration (FDA), inspect individual complexes, and manually redock the ligand using VR controllers (Figure 3). In the end, they are invited to explore a SARS-CoV-2 PetWorld model,^23^ learning how to traverse mesoscale models using teleportation to avoid motion sickness (Figure 4).

### Additional Features for Molecular Modeling

To be of real practical value, the virtual reality interface described here must be combined with a large number of molecular modeling functions so that useful scientific work can be done that extends far beyond visual exploration. Of course all these functions can be used without a VR headset too, so YASARA Model is helpful for everyone interested in molecular modeling.

**Figure 5:**
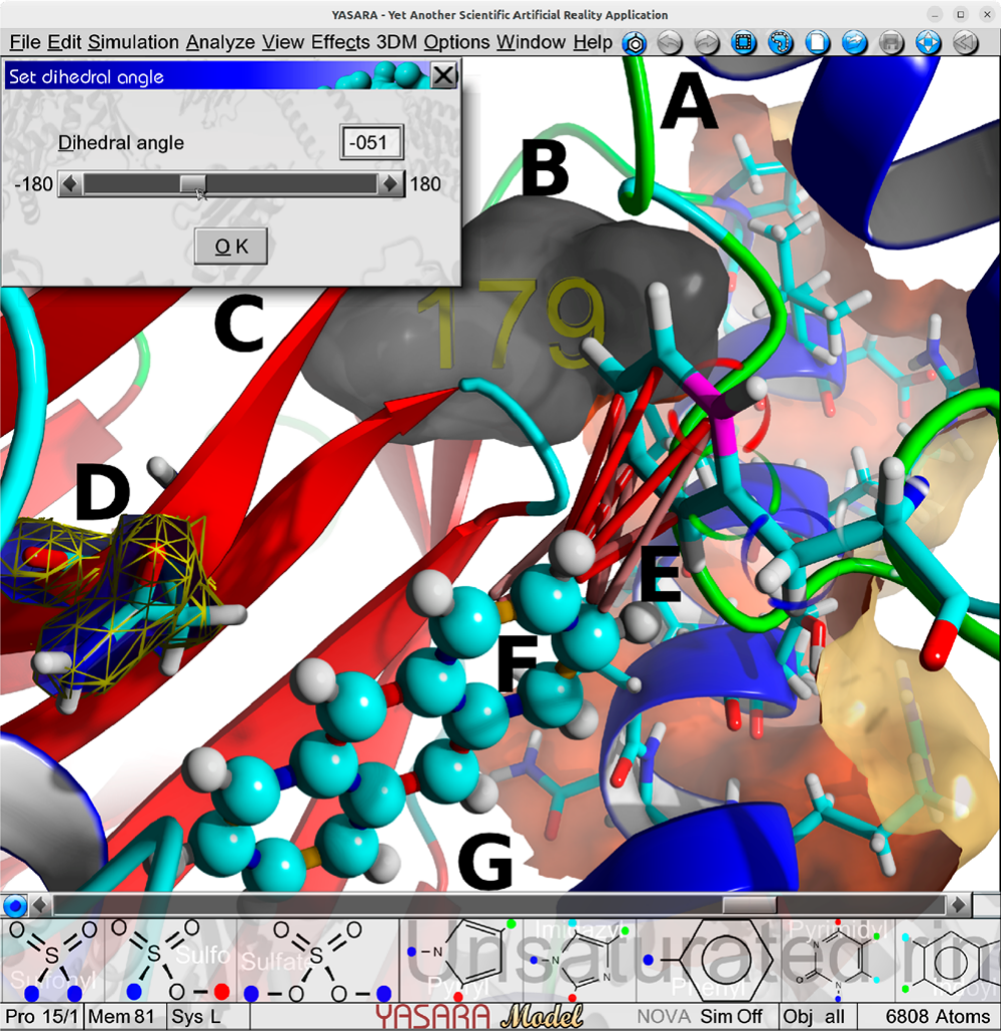
A selection of YASARA Model features exemplified by the active site of naphthalene 1,2-dioxygenase bound to anthracene (PDB ID: 2HMM). (A) Orange-colored partial molecular surface of the α-helix spanned by residues 337–352. (B) Cavity of 179 Å^3^ volume, labeled with 3D letters, bounded by an internal molecular surface colored gray. (C) Interactively rotating the Cα-Cβ-Cγ-Cδ1 dihedral angle of Phe 224 marked with colored fireflies on the mid right side. (D) Electron density contour surface of Asn 297 loaded from the RCSB and shown as a yellow wire frame. (E) T-shaped π-π interactions depicted as lines between Phe 224 and the ligand. (F) Resonance bonds of anthracene colored by fractional bond order (blue: 1.25, red: 1.5, orange: 1.75). (G) Some functional groups of the interactive small molecule builder. Clicking the colored dot joints in the menu will add the respective group using the selected joint.

On top of the features described previously for YASARA View,^24^ which are all included in YASARA Model, we implemented several additional functionalities to aid molecular modelers: van der Waals, molecular, and solvent-accessible surfaces can be shown and calculated (including their volume), not only for entire objects but also partially by first defining a surface environment (i.e., the atoms that form a surface) and then extracting the surface of a subset (Figure 5A). Cavities formed by these surfaces are shown, and their volume can be calculated (Figure 5B). Distances, angles, and dihedrals can be changed interactively (Figure 5C), while visible surfaces adapt in real-time, thanks to highly optimized algorithms. Measuring these geometric features is possible not only between individual atoms but also between groups of atoms, allowing one to calculate, e.g., the angle between two α-helices. Geometry errors like cis-peptide bonds or wrong stereoisomers can be detected. Large protein complexes are built by joining or replicating single proteins, using, for example, the information about the biologically relevant oligomer or the crystal packing provided in PDB files. Electron densities and other maps in many formats can be loaded, visualized in many ways (Figure 5D), modified, and exported again. Contacts and interactions such as hydrogen bonds and hydrophobic or π-π interactions are shown and analyzed (Figure 5E). Since small-molecule ligands are often the most important part of a structure, YASARA Model provides several functions to deal with them: based on a user-defined pH and a large library of p*K*_a_ values, protonation states and fractional bond orders are assigned (Figure 5F). An interactive small-molecule builder with more than 50 functional groups helps to quickly modify ligands or build them from scratch (Figure 5G). Small molecules can be aligned by superposing them on the largest common fragment. To help with large data sets and automation, YASARA Model supports batch processing and is fully scriptable, using either Python or Yanaconda, the built-in simple macro language, which allows one to directly record user input as a macro. Research results can be stored in tables, visualized, and imported into spreadsheet software. Presentation functions are available to animate molecules, text with 3D letters (Figure 5B), images, and texture-mapped 3D models and encode the resulting animations in MPEG4 format. A large number of such interactive movies are provided as eLearning tutorials.

## Conclusion

*In silico* molecular modeling has over the last 50 years proven to be an invaluable tool in biomedical research. For a molecular modeling software package like YASARA, which has been in active development for decades and accrued a significant userbase, the integration of new technology like VR headsets always has to be carefully weighted against possible disruption of established workflows. When we tried the first VR headsets with controllers, we were immediately fascinated by the completely new level of immersion and interaction. While 3D glasses offer a view through a window, VR allows us to walk through the door, initiating billion dollar investments from companies like Meta and Microsoft. But we also noted the main drawback: as soon as the first fascination is gone and hours of hard modeling work lie ahead, one feels the desire for the seated, relaxing workplace known from 3D glasses. After three man-years of low-level work on VR graphics, user interfaces, and headset cameras to optimally include mouse and keyboard, our conclusion is that comparably relaxing work in VR is possible. While the headset is heavier and thus less convenient than 3D glasses, this is compensated for by the ability to freely look around and grab objects. The amount of compensation is certainly subjective, but for us it is so high that we have not used 3D glasses since. Users already accustomed to working with shutter glasses can now easily transition to VR and profit from future technological advances heavily supported by the video gaming industry. To ease the transition, we provide an extensive tutorial, guiding the user from the basic setup to integrating VR into their drug design and modeling pipelines. The many general molecular modeling features are practical tools to aid scientists in and outside VR.

Our next step is to bring interactive molecular dynamics to VR, allowing one to push and pull individual atoms or whole molecules during live simulations. This feature has already been partially implemented as part of YASARA Dynamics.^25^

## Data Availability

An introductory video can be viewed at www.YASARA.org/vr. Our implementation is freely available for academic users as part of the YASARA Model program from www.YASARA.org. Free updates and support will be provided for at least two years after publication. Models like the SARS-CoV-2 structure (Figure 4) can be downloaded from www.YASARA.org/petworld, a Creative Commons platform for sharing giant biomolecular structures. Interactive tutorials, including the VR tutorial, can be found as open source YASARA macros at www.YASARA.org/movies.
